# Impact of index of multiple deprivation and ethnicity on breast screening uptake in the North West of England

**DOI:** 10.1186/bcr3786

**Published:** 2015-11-05

**Authors:** Jayeshwaraj Bhola, Anil Jain, Philip Foden

**Affiliations:** 1 The University of Manchester, UK; 2 The Nightingale Centre and Genesis Prevention Centre, University Hospital of South Manchester NHS Foundation Trust, Manchester, UK

## Introduction

The aim was to investigate the impact of index of multiple deprivation (IMD) and ethnicity on breast cancer screening uptake in the North West of England.

## Methods

Data for screening uptake rates were collected from 2005 to 2014 using data from the North West Breast Screening Units and the annual breast screening statistics reports. These were correlated with the IMD published in 2007 and 2010. The uptake rates were also correlated with ethnicity data obtained from the Census 2011. Then, the results for ethnicity were adjusted for IMD.

## Results

Both prevalent and incident uptake rates have declined from 2005/06 to 2013/14. Deprivation was shown to negatively correlate with breast screening uptake in all rounds, the strongest correlation being with prevalent screening rounds (IMD 2007 *p *= 0.005 and 2010 *p *= 0.016). The incident round negative correlation was IMD 2007 *p *= 0.002 (significant) and IMD 2010 *p *= 0.163 (not significant). For ethnicity, the Caucasian population showed a positive correlation while Asian, a negative correlation. This was more significant in the Pakistani and Bangladeshi groups. Interestingly, when the results were adjusted for deprivation, ethnicity did not show a significant correlation with uptake rates.

## Conclusions

Our results clearly show that the more deprived an area, the lower the breast screening uptake rate. Moreover, the higher the proportion of Asian in a population, the lower the uptake rates and this is more significant in the Pakistani and Bangladeshi group compared to the Indian and Chinese. Overall the impact is most marked in the prevalent round.

